# 

**Published:** 1856-07

**Authors:** 


### CONTENTS.

***ORIGINAL COMMUNICATIONS.*** PAGE

ARTICLE I.—The Diseases, etc., of the Bible. By Dr. W. T. GRANT, of Wrightsboro', Columbia County, Georgia, .......... 641

ARTICLE II.—Viratrum Veride. By ISHAM H. RAGAN, M. D., of Lithonia, Georgia, .......... 666

***SELECTIONS.***

Annual Address of Prof. GEO. B. WOOD, to the American Medical Association, upon leaving the Chair, .......... 670

Translations from Foreign Journals. By CH. F. J. SEHLBACH, M. D., Newark, New Jersey, .......... 678

Probable Fracture of Base or Scull from a Fall. By ABRAHAM LIVEZEY, A. M., M. D., Lumberville, Penn., .......... 680

A Chapter of Accidents. By R. THOMPSON, M. D., of Nashville, Tenn. .......... 683

***EDITORIAL AND MISCELLANEOUS.***

Atlanta Medical College .......... 686

Bibliographical .......... 688

New Orleans School of Medicine, .......... 691

Errata .......... 691

Subscriptions Received, .......... 691

Abstract of the Proceedings of the Ninth Annual Session of the American Medical Association, held in Detroit, May 6, 7, 8, and 9, 1856 .......... 691

